# Effects of hand-press pellet on pain and daily life of elders with chronic lower back pain: randomized controlled trial

**DOI:** 10.1186/s12906-024-04481-7

**Published:** 2024-05-03

**Authors:** Hyojung Park, Hyejin Lee

**Affiliations:** Ewha Womans University, 52, Ewhayeodae-gil, Seodaemun-gu, Seoul, 03760 South Korea

**Keywords:** Hand pressed pellet, Chronic low back pain, Pain pressure threshold

## Abstract

**Background:**

For elderly people with chronic lower back pain who need long-term management, there is a need for a nursing intervention study that is effective, is easy to perform, and applies complementary and alternative therapies to manage pain without repulsion. Hand pressure therapy is a treatment indigenous to Korea used to reduce pain and improve functions of daily life by applying acupuncture, pressure sticks, and moxibustion to parts of the hand as they relate to parts of the body. This research is to identify the effects of pellet pressed on the hand on pain and the daily lives of elders with chronic lower back pain (CLBP).

**Methods:**

The hand pressed-pellet intervention period was six weeks long. Twenty-seven patients in the intervention group and twenty-four patients in the placebo control group were recruited from elderly over sixty-five who used welfare centers. In the intervention group, hand pressed-pellet therapy was conducted in eleven acupressure response zones related to CLBP, and the placebo control group was provided with similar therapy and zones, but unrelated to CLBP. The research tool measured the intensity of CLBP using the Visual Analogue Scale (VAS), the Korean Owestry Disability Index (K-ODI), which are subjective indicators, and the Compact Digital Algometer, which is an objective indicator.

**Result:**

The pain intensity (VAS) measured after six weeks of hand pressed-pellet therapy showed significant difference between the two groups compared to their pain before the experiment (F = 60.522, *p* < .001). There was a significant difference between the two groups in the pain pressure threshold using pressure statistics (F = 8.940, *p* < .001), and in CLBP dysfunction evaluation index (K-ODI) after applying pressed pellet to the hand (Z = − 3.540, *p* < .001).

**Conclusion:**

Subjective indicators were measured to verify the effect of hand pressed-pellet therapy on CLBP, and the result confirmed that the hand pressed-pellet therapy was effective in alleviating CLBP.

**Trial registration:**

The study was registered retrospectively with reference number KCT0008024 on 23/12/2022.

## Introduction

Recent research indicates that the global incidence of low back pain (LBP) is on the rise, driven by the effects of aging and population growth. In 2020, approximately 619 million individuals worldwide were suffering from LBP, with projections estimating an increase to 843 million by 2050 [1]. LBP is a leading cause of disability across all age groups globally, but the increase in LBP cases is expected to be particularly significant in the Asian region due to aging [2]. Low back pain in the elderly acts as a significant limiting factor for physical activities in later life, leading to changes in daily living that can cause social isolation, psychological disorders, and cognitive impairments [3, 4]. Low back pain is characterized by pain and discomfort located between the rib cage’s edge and the lower buttock crease, regardless of the presence of leg pain. It is categorized based on duration into acute (less than 6 weeks), subacute (6 to 12 weeks), and chronic (more than 12 weeks) stages [1]. Notably, low back pain in the elderly is often attributed to degenerative joint changes and hormonal alterations associated with aging, increasing the likelihood of the pain becoming chronic and persisting for more than three months [5]. Drug therapy, surgery, and nondrug therapy are generally used to solve the problem of lower back pain in elderly people. However, management of lower back pain through drugs and surgery is limited because it has the potential to lead to tolerance, dependence, and abuse, and various side effects may result [6, 7]. The American College of Physicians has published guidelines for treating lower back pain that recommend that nondrug therapies such as exercise, rehabilitation, acupuncture, and meditation be preferred over unnecessary tests and drug treatment for chronic lower back pain [8]. Complementary and alternative therapies are increasing in demand and use in relation to problems that are difficult to solve with drug therapy or surgery [7, 9]. Therefore, for elderly people with chronic lower back pain who need long-term management, there is a need for a nursing intervention study that is effective, is easy to perform, and applies complementary and alternative therapies to manage pain without repulsion.

Hand pressure therapy is a treatment indigenous to Korea used to reduce pain and improve functions of daily life by applying acupuncture, pressure sticks, and moxibustion to parts of the hand as they relate to parts of the body [10]. These methods are easy to access and simple to apply, and their effectiveness is currently being proven through studies on pain reduction using hand-press therapy [11–14]. Hand pressure therapy is distinguished by its non-invasive approach, utilizing the principles of reflex therapy to exert effects on both the peripheral and central nervous systems. Stimulation of acupoints on the hand activates nerve fibers, which in turn engage central nervous system components like the Periaqueductal Gray, Rostral Ventromedial Medulla, and the descending inhibitory pathways. This interaction serves to suppress the transmission of pain signals within the spinal cord, thereby offering an analgesic effect. Moreover, hand pressure therapy facilitates the release of neurotransmitters such as endorphins and serotonin, which play a crucial role in the body’s natural pain management system. The repetitive nature of this stimulation can lead to the reorganization of pain-related neural circuits, potentially restoring the impaired pain control system often observed in individuals suffering from chronic pain [15–17]. Therefore, it represents a therapeutic approach capable of reducing chronic low back pain in the elderly and manifesting changes in daily life activities.

A number of studies on the effects of hand pressed-pellet therapy have been done [13, 14, 18–21], but studies targeting the elderly are limited, and most of the studies are in combination with other therapies rather than the hand pressed-pellet intervention alone. In addition, since these studies measured only subjective pain intensity in relation to pain, or because there was no intervention for the control group, there are limits in verifying the objective numerical change in pain intensity, the sole effect of the hand pressed-pellet therapy, and the direct effect on the change of daily life.

Therefore, this study hypothesized that the intervention group receiving hand pressure therapy would experience a reduction in pain intensity, an increase in pain threshold, and an improvement in daily activities compared to the placebo control group. Accordingly, this study is divided into an intervention group and a placebo group, and using subjective and objective indicators, the effect of hand pressed-pellet therapy on back pain in elderly people is studied through the sole mediation of hand pressed-pellet therapy.

## Materials and methods

### Study design

This study is experimental research using randomized controlled trial to identify the effects of hand pressed-pellet therapy on pain intensity, pain pressure threshold, and daily activities of elderly people complaining of chronic lower back pain.

### Participants and randomization

This study was conducted from November 1, 2021, to January 30, 2022. Subjects using four elderly welfare facilities in A City, B City, S City, and I City were selected. The selection criteria were elderly people who were 65 years of age or older, those who complained of lower back pain for more than three months, those who could communicate and respond to questionnaires, and those who had no experience in hand pressed-pellet therapy. Exclusion criteria included those who were using other complementary and alternative therapies to relieve lower back pain, whose lower back pain was caused by cancer or a fracture, who had trauma or lesions on the hands, who had allergies, and who were currently receiving treatment for back pain.

**Fig. 1:**
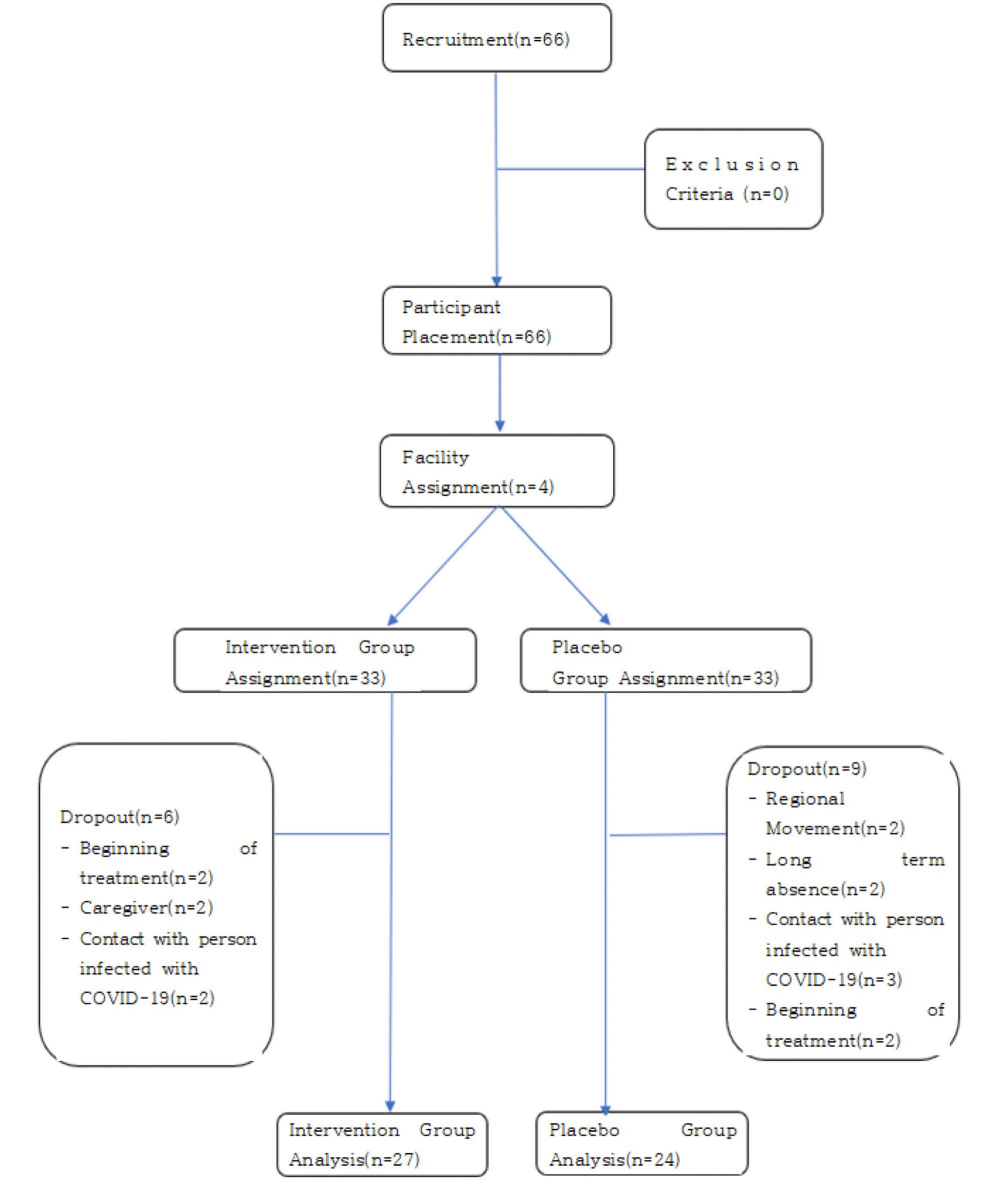
Participant flowchart

The sample size for this study was calculated using the G*power 3.1 program. To calculate the sample size, we conservatively set the effect size to 0.7 based on the effect size reported in a previous study [13], which corresponds to a medium to large effect size according to the criteria presented by Cohen [22]. Additionally, the Type I error (α) was set at 0.05, and the power (1-β) was set at 0.8, which are commonly used standards in research [23], taking into consideration a balanced approach to the possibility of false positives and false negatives. A one-tailed t-test was used to calculate the necessary number of samples for an independent samples t-test. As a result, it was found that a total of 52 participants, with 26 participants in each of the experimental and placebo control groups, were needed. With an expected dropout rate of 20%, considering the coronavirus situation and the study period of six weeks, thirty-three participants were placed in the intervention group and thirty-three were placed in the placebo control group, for a total of sixty-six patients. Each facility randomly assigned participants to two groups using an Excel program. Each with thirty-three participants in the intervention group and thirty-three in the placebo control group. During the study, six patients in the intervention group and nine patients in the placebo control group dropped out due to coronavirus-related or personal circumstances. A total of fifty-one participants, twenty-seven in the intervention group and twenty-four in the placebo control group, were used to analyze the study results [Fig. 1].

### Intervention

For the intervention of the pressed pellet, a silver-colored Seoambong (aluminum patch) product made of aluminum with small bumps the size of grains was used. Based on previous research [11–13, 24], the mediation was conducted once a week for a total of six times.

**Fig. 2:**
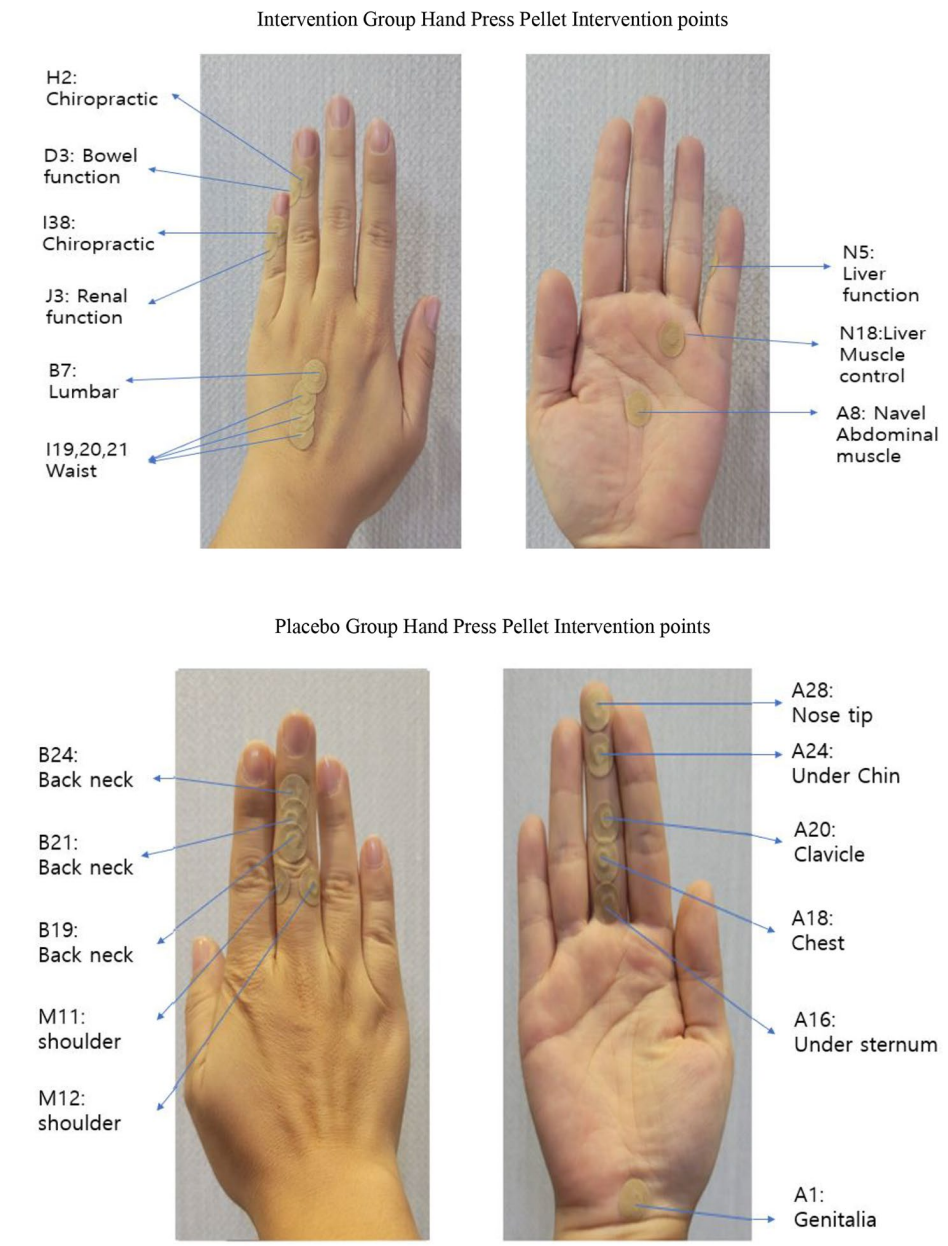
Hand- press points used in intervention group and control group

The researcher directly applied the pressed-pellet therapy to both hands based on the evidence that it is effective to do so. For the intervention group, the pressed-pellet therapy was applied at a total of eleven sites: D3, regulating blood for colon function; N5, for regulating liver function; J3, for regulating kidney function; I19/I20/I21 and N18, for muscle strengthening; A8, umbilical/abdominal muscle maintenance acupuncture points; H2, chiropractic acupuncture points directly related to lower back pain; I38, chiropractic points directly related to back pain and B7, lumbar vertebrae [Fig. 2]. In addition, the control group was also mediated with placebo hand pressed-pellet therapy. For the control group, the mediation was applied to a total of eleven sites that are unrelated to lower back pain: A28, nose tip; A24, under the chin; A20, Clavicle; A18, center of chest; A16, under the genitalia; A1, genitalia; B24/B2/B19, back of neck; and M11-2, shoulder blood duct [Fig. 2]. It was possible to maintain four hours of pressure or more [10, 25] when attaching the Seoambong, and the blood flow stimulated by the hand pressed pellet is shown in Fig. 2.

For the preliminary investigation, the intervention group and the placebo control group were asked about general and health- and disease-related issues, pain intensity (using the visual analog scale, or VAS), pain level, personal care, transfer, walking, sitting, sleeping, standing, social activities, going out, and so on. The investigator used a questionnaire tool (K-ODI) related to activities of daily living. After participants had completed the questionnaire in the welfare facilities, the investigator measured the pain pressure threshold of the participants in the corresponding facilities. Every week, before the hand pressed-pellet intervention was performed, the research assistant checked the pain intensity, and then the investigator reviewed the discomfort and side effects at the intervention site, measured the pain pressure threshold, and performed the hand pressed-pellet intervention. One week after the end of the six hand pressed-pellet interventions, the research assistant surveyed the pain intensity (VAS) and daily living activity measurement tool (K-ODI) in the same way as the pre-examination method, and the investigator measured the pain pressure threshold and conducted a post-mortem survey.

### Instruments

#### VAS

In this study, *pain intensity* refers to the degree of pain the subject feels using the visual analog scale (VAS). A score of 0 indicates no pain, and a score of 10 indicates severe pain; overall, a lower score indicates less pain.

#### PPTs

In this study, pain pressure threshold was measured using a pressure painometer, specifically, the Compact Digital Algometer (pain test EPX25 Algometer, Wagner Instrument, USA). PPT is defined as the minimum amount of pressure necessary to elicit pain or discomfort. The pressure is applied perpendicularly to the skin at a constant rate of increase until the participant reports the initial sensation of pain. The pressure at which pain is first perceived is recorded as the PPT value, expressed in pounds per square inch (Ibf). In this study, PPT measurements were conducted at the site of maximal pain in the lumbar area, with two measurements taken at each assessment point and a one-minute interval between measurements to determine the average value [26, 27].

#### K-ODI

In this study, daily activities were assessed using the Korean Oswestry disability index (K-ODI) developed by Fairbank et al. [28] (1980) and modified and supplemented by C. Jeon et al. [29] to include pain level, walking, sitting, sleeping, standing, sleeping, social activities, and going out. The lower the total score of the nine items related to daily life behaviors was, the less problem participants had with daily life activities.

### Data collection

This study was conducted after receiving approval from the E University Bioethics Committee (IRB NO. ewha-202110-0020-01). After seeking cooperation from the managers of four elderly welfare facilities in A city, B city, S city, and I city in Korea, a notice was posted in each facility to recruit participants. Subjects who voluntarily consented were given informed and written consent, and both researchers and subjects were required to strictly observe quarantine rules such as hand hygiene and wearing masks to prevent the spread of COVID-19.

The investigator directly applied the hand pressed-pellet therapy to the hands of the intervention group and placebo group participants for each study. This experiment was applied while the intervetion group and placebo group did not know which group they were assigned to. (single blined)There were no disadvantages to the participants who withdrew from the study. Furthermore, the hand pressed-pellet mediation is known as a safe measure with less pain and free from side effects. The contact number of the investigator was provided to the participants for any inquiry regarding discomfort or questions about the study.

### Data analysis

The collected data were analyzed using IBM SPSS Statistics version 26.0.

The specific analysis method is as follows.

1. The general characteristics and health-related characteristics of the intervention group and the placebo control group were analyzed with descriptive statistics using the mean, standard deviation, frequency, and percentage.
2. The prior homogeneity of general characteristics and health-related characteristics between the intervention group and the placebo control group was analyzed by the independent *t*-test, Fisher’s exact test, and Chi-square test.
3. The normality verification of the dependent variable (VAS, pain pressure threshold, K-ODI) before the intervention of the intervention group and the placebo control group was analyzed by the Shapiro-Wilk test, and a nonparametric statistical technique was used for the variable that did not follow the normal distribution.
4. The intragroup and intergroup differences and changes over time of the outcome variables of the intervention group and the placebo control group were analyzed using generalized estimating equations, repeated measures ANOVA, Wilcoxon test, Mann–Whitney U test, paired *t*-test, and independent *t*-test.

## Results

### Participant characteristics

The general characteristics of the study participants were pre-examined through questionnaires, including gender, age, marital status, cohabitation status, educational background, and experience with hand pressed-pellet therapy. An independent *t*-test, Fisher’s exact test, and a Chi-square test were conducted to verify prior homogeneity between the intervention group and the placebo control group. As a result of verifying the homogeneity of the general characteristics of the two groups, there was no significant difference in any category with the significance level of 0.05, confirming that the intervention group and the placebo control group were homogenous groups (see Table 1).

**Table 1:** General characteristics of participants (*N* = 51)

		I(*n* = 27)	P(*n* = 24)		
Characteristics	Variable	n(%) or Mean ± SD		x^2^ or t	*p*
Age		75.37 ± 7.40	72.46 ± 8.52	-1.306	0.198
Gender	Female	26(96.3)	22(91.7)		0.595†
	Male	1(3.7)	2(8.3)		
Marriage	Married	12(44.4)	12(50)	0.157	0.692
	By death /Devorced	15(55.6)	12(50)		
Roommate	Yes	13(48.1)	15(62.5)	1.057	0.400
	No	14(51.9)	9(37.5)		
	Ignorance	10(37)	5(20.8)		0.060†
Educational Bacground	Elementary	9(33.3)	3(12.5)		
	Middle	4(14.8)	6(25)		
	High	4(14.9)	8(33.3)		
	College or higher	0(0)	2(8.4)		
Disease duration		5.37 ± 3.46	6.55 ± 5.94	0.880	0.383
Treatment experiment	Yes	23(85.2)	16(66.7)		0.187†
	No	4(14.8)	8(33.3)		
Back surgery	Yes	5(18.5)	2(8.3)		0.425†
	No	22(81.5)	22(91.7)		
Physical therapy	Yes	13(48.1)	12(50)	0.017	> 0.999
	No	14(51.9)	12(50)		
Herbal treatment	Yes	5(18.5)	2(8.3)		0.425†
	No	22(81.5)	22(91.7)		
Hypertension	Yes	10(37)	8(33.3)	0.076	> 0.999
	No	17(63)	16(66.7)		
Diabetes	Yes	6(22.2)	3(12.5)		0.473†
	No	21(77.8)	21(87.5)		
Bone joint disease	Yes	4(14.8)	1(4.1)		0.354†
	No	23(85.2)	23(95.9)		
Other	Yes	2(7.4)	1(4.1)		> 0.999†
	No	25(92.6)	23(95.9)		

† Fisher’s exact test

I, intervention group; P, placebo control group; SD, Standard deviation

The health-related characteristics of the study participants were also pre-examined through questionnaires, including the duration of back pain, past treatment experience of lower back pain, types of treatment experience, and currently diagnosed diseases. An independent *t*-test, Fisher’s exact test, and a Chi-square test were performed to verify prior homogeneity between the intervention group and the placebo control group. As a result of prior homogeneity verification of the health-related characteristics of the two groups, there was no significant difference in any category with a significance level of 0.05, confirming that the intervention group and the placebo control group were homogeneous groups (see Table 1).

### Result of VAS

The hypothesis stating “The intervention group who received hand pressed-pellet therapy related to lower back pain would have reduced pain intensity compared to the placebo control group” was supported. Pain intensity showed a significant difference between the two groups according to whether or not the hand pressed-pellet mediation had been performed and over time (F = 60.522, *p* < .001) (see Table 2), and Table 3 presents the results of comparing changes in pain intensity, measured by the VAS, over time between the intervention group, which received spine compression therapy, and the placebo control group. This table illustrates the changes in pain intensity from the baseline at each subsequent week. A statistically significant difference first appears at the third week after the intervention, where the intervention group shows a significant decrease in pain intensity compared to the baseline (t = − 4.388, *p* < .001), suggesting the effectiveness of the intervention in alleviating pain. Similar patterns are observed in the 4th, 5th, and 6th weeks, indicating the sustained effect of the treatment.

**Table 2:** Changes in the intensity of low back pain (VAS) between the intervention group and the control group (*N* = 51)

	I(*n* = 27)	P(*n* = 24)	Source†	F†	*p*†
VAS	Mean ± SD				
At baseline	5.37 ± 2.20	4.88 ± 2.23	G	0.247	0.619
After 1 Weeks	5.19 ± 2.18	4.96 ± 2.27			
After 2 Weeks	4.78 ± 2.15	4.79 ± 2.17	T		< 0.001
After 3 Weeks	4.40 ± 2.00	4.75 ± 2.17			
After 4 Weeks	4.33 ± 1.95	4.83 ± 2.12			
After 5 Weeks	4.04 ± 1.95	4.92 ± 2.12	G x T	60.522	< 0.001
After 6 Weeks	4.04 ± 1.95	4.93 ± 1.97			

† Generalized Estimating Equation

G, group; G x T, group x time; I, intervention group; P, placebo control group; SD, Standard deviation; T; time; VAS, visual Analogue Scale

**Table 3:** Difference in VAS change between intervention group and control group (*N* = 51)

VAS	Group	Variance^+^	In the Group		Between the Groups	
		Mean ± SD	t	*p*	t	*p*
After 1 Wk– at baseline	I	-0.19 ± 0.40	-2.236	0.063	-1.471	0.141
	*P*	0.08 ± 0.72	-0.577	0.781		
After 2 Wk – at baseline	I	-0.41 ± 0.50	-3.771	< 0.001	-1.229	0.219
	*P*	-0.17 ± 0.82	-0.707	0.727		
After 3 Wk – at baseline	I	-0.96 ± 0.52	-4.564	< 0.001	-4.388	< 0.001
	*P*	-0.13 ± 0.61	-1.000	0.508		
After 4 Wk – at baseline	I	-1.04 ± 0.59	-4.460	< 0.001	-4.654	< 0.001
	*P*	-0.42 ± 0.69	-0.302	< 0.999		
After 5 Wk – at baseline	I	-1.33 ± 0.73	-4.332	< 0.001	-4.955	< 0.001
	*P*	0.04 ± 0.75	-0.277	< 0.999		
After 6 Wk – at baseline	I	-1.33 ± 0.73	-4.332	< 0.001	-4.791	< 0.001
	*P*	-0.42 ± 0.75	-0.277	< 0.999		

^+^After X week(s) – at baseline

I, intervention group; P, placebo control group; SD, Standard deviation; VAS, visual Analogue Scale

### Results of pain pressure threshold

The hypothesis stating, “The intervention group receiving hand pressed-pellet therapy related to lower back pain will have an higher pain pressure threshold than the placebo control group” was supported. The pain pressure threshold between the two groups showed a significant difference according to whether or not the hand pressed-pellet mediation was performed and how much time had passed (F = 8.940, *p* < .001) (see Table 4), and Table 5 compares the changes in PPT between the intervention group, which received spine compression therapy, and the placebo control group. This table reports the amounts of change from the baseline at 1 week, 2 weeks, 3 weeks, 4 weeks, 5 weeks, and 6 weeks post-intervention. The analysis reveals a significant increase in the PPT in the intervention group from the first week following treatment (t = − 2.892, *p* = .006), indicating a decreased sensitivity to pain in the intervention group compared to the control group. This difference persists over time, with a significant difference still observable at the 6th week (t = − 4.702, *p* < .001). This demonstrates the therapy’s effectiveness in enhancing pain tolerance capacity.

**Table 4:** Changes in pain pressure threshold between intervention group and control group (*N* = 51)

Pressure Threshold	I(*n* = 27)	P(*n* = 24)	Source	F	*p*
	Mean ± SD				
At baseline	7.28 ± 3.45	8.08 ± 3.00	G	0.051	0.822
After 1 Weeks	7.77 ± 3.29	8.05 ± 2.88			
After 2 Weeks	8.47 ± 3.72	8.19 ± 2.75	T	13.096	< 0.001
After 3 Weeks	8.54 ± 3.43	8.21 ± 2.88			
After 4 Weeks	8.58 ± 3.41	8.35 ± 2.76			
After 5 Weeks	9.01 ± 3.38	8.26 ± 2.96	G*T	8.940	< 0.001
After 6 Weeks	8.98 ± 3.34	8.11 ± 2.95			

G, group; G x T, group x time; I, intervention group; P, placebo control group; SD, Standard deviation; T; time

**Table 5:** Difference in the amount of change in pain pressure threshold between the intervention group and the control group (*N* = 51)

Pain Threshold	Group	Variance^+^	In the Group		Between the Groups	
		M ± SD	t	*p*	t	*p*
After 1 Wk – at baseline	I	-0.49 ± 0.80	-3.172	0.004	-2.892	0.006
	*P*	0.03 ± 0.39	-0.395	0.696		
After 2 Wk – at baseline	I	-1.19 ± 1.83	-3.397	0.002	-2.660	0.011
	*P*	-0.10 ± 0.89	-0.563	0.579		
After 3 Wk – at baseline	I	-1.26 ± 1.61	-4.061	< 0.001	-3.184	0.003
	*P*	-0.13 ± 0.70	-0.887	0.384		
After 4 Wk – at baseline	I	-1.30 ± 1.51	-4.470	< 0.001	-3.064	0.004
	*P*	-0.27 ± 0.71	-1.822	0.082		
After 5 Wk – at baseline	I	-1.73 ± 1.80	-5.014	< 0.001	-4.064	< 0.001
	*P*	-0.18 ± 0.57	-1.532	0.139		
After 6 Wk – at baseline	I	-1.70 ± 1.73	-5.108	< 0.001	-4.702	< 0.001
	*P*	-0.03 ± 0.18	-0.820	0.421		

^+^After X week(s) – at baseline

I, intervention group; P, placebo control group; SD, Standard deviation

### Result of K-ODI

The hypothesis stating, “The intervention group receiving hand pressed-pellet therapy related to lower back pain will have improved activities of daily living compared to the placebo control group” was supported. The post-mortem K-ODI score of the intervention group was significantly decreased (Z = − 4.025, *p* < .001), and the difference between the two groups after the completion of the intervention of the hand pressed-pellet therapy was also statistically significant (Z = − 3.540, *p* < .001) (see Table 6).

**Table 6:** Changes in low back pain-related impairments in activities of daily living in the intervention and control group (*N* = 51)

	Group	Pre intervention	Post intervention	Variance^+^	In the Group		Between the Groups	
		Mean ± SD	Mean ± SD	Mean ± SD	Z*	P*	Z†	p†
K-ODI	I	18.33 ± 7.67	14.82 ± 4.98	-3.52 ± 3.14	-4.025	< 0.001	-3.540	< 0.001
	*P*	15.21 ± 6.51	14.54 ± 6.19	-0.67 ± 1.09	-2.800	0.005		

* Wilcoxon test; † Mann-Whitney U test; ^+^ After intervention at baseline

I, intervention group; K-ODI, Korean Oswestry Disability Index; P, placebo control group; SD, Standard deviation

## Discussion

The purpose of this study was to verify the effect on pain intensity, pain pressure threshold, and activities of daily living achieved by applying hand pressed-pellet therapy for a total of six weeks to elderly people complaining of chronic lower back pain. The result of this study shows that hand pressed-pellet therapy was effective in reducing back pain and improving activities of daily living in elderly people with chronic lower back pain. In this study, the subjective pain score on the VAS decreased statistically and significantly in the intervention group compared to the placebo control group over time. These outcomes supported the results of this study corresponding to previous research that reported, “It is effective in reducing pain after applying the hand press pellet therapy to a subject with low back pain” [11, 12, 18, 24]. However, unlike this study, previous studies did not apply any mediation to the control group. In this study, a difference was detected between the intervention group and the control group even after placebo mediation. Therefore, it can be confirmed that the hand pressed-pellet therapy applied in previous studies and the current study is an effective nursing mediation method for pain control.

In this study, participants’ VAS scores for lower back pain decreased after three weeks of hand pressed-pellet mediation. A study on the effects of other complementary and alternative therapies for lower back pain showed that the VAS score started to decrease from week six in the case of aural pressure therapy [30] and from week twelve in the case of lumbar moxibustion therapy [31]. These results show that hand pressed-pellet therapy not only reduces pain quickly but also has a lasting effect. Also, Hand pressed-pellet therapy afferent nerve fibers by physically stimulating specific acupoints, which then stimulates the central nervous system’s PAG, RVM, and descending inhibitory pathways [16, 17]. The activation of these pathways inhibits the transmission of pain signals in the spinal dorsal horn, producing an analgesic effect. In this study, the experimental group that applied hand acupressure showed a statistically significant decrease in VAS scores over time compared to the placebo control group, suggesting that hand acupressure is effective in reducing pain. Therefore, hand pressed-pellet therapy can be used as a nursing mediation for pain reduction not only for those with chronic lower back pain but also for those with acute pain.

In a study in which an adult male with lower back pain was treated with the hand pressed-pellet mediation, the mediation on the H2, I38, I19, and A8 blood (which overlaps with this study) sites reduced lower back pain. In a study in which the hand pressed-pellet therapy was conducted with patients suffering from chronic lower back pain, the pain was reduced by mediation on the H2, I38, B7, I21, I19, and A8 blood sites as well [12, 18, 24]. The acupoints common to the present study and previous studies related to lower back pain were H2 and I38. Therefore, it is important to include the H2 and I38 chiropractic points in the mediation of hand pressed-pellet therapy to reduce pain in patients with lower back pain.

Most previous studies used subjective pain indicators, including the VAS, to evaluate the pain intensity of subjects with lower back pain [18, 23, 30]. Subjective indicators make it easy to collect data, are adequate for quickly judging pain information, and are often used because of their practicality. However, VAS, a pain-collection tool based on a person’s judgment, is a subjective evaluation tool and is limited in its ability to evaluate the validity of a reported pain level. On the other hand, the pressure painometer is an acceptable objective tool to measure the pain pressure threshold even after the lapse of time in the intervention because there is no difference in measurement by different inspectors and it is not affected by time [32]. In this study using an objective tool, the pain pressure threshold for lower back pain increased continuously after the mediation, and a difference from the placebo control group appeared at one week after the mediation. While the subjective pain index, the VAS, showed a decrease after three weeks, the objective index showed that back pain was effectively reduced within a short period of time. This indicates that hand acupressure promotes the secretion of neurotransmitters such as endorphins, enkephalins, and serotonin, thereby activating the endogenous analgesic system [16]. Serotonin, in particular, plays a crucial role in regulating the transmission of pain at the spinal level through descending inhibitory pathways. In this study, the pain pressure threshold of the lower back area measured using a pressure algometer continuously increased after the application of hand acupressure, with differences from the placebo control group appearing one week after intervention. This demonstrates that hand acupressure shows effectiveness in objective pain indicators in a short period. But, since there are no previous studies comparing the pain pressure threshold over time using a pressure painometer in a study that performed the hand pressed-pellet mediation, there are no other results available for direct comparison. Therefore, repeated studies related to lower back pain using a pressure painometer will be needed.

The result of this study showed an effect of reducing pain intensity, increasing pain pressure threshold, and improving activities of daily living in the mediation group, which was measured weekly after the hand pressed-pellet therapy intervention. Between the two groups, the change in pain intensity was different at three weeks after the mediation and the change in pain pressure threshold was different one week after the mediation. The result from previous research in which hand pressed-pellet therapy was applied to elderly participants with lower back, knee, and joint pain showed that the VAS score decreased after week two and week four [11], similar to the results of the current research in which the VAS score decreased from three weeks after the hand pressed-pellet mediation. However, the previous research conducted only interim and post-mortem surveys rather than weekly surveys, and most other previous studies [12, 18, 24] conducted only post-mortem measurements, which differs from this study. Therefore, it was confirmed that the hand pressed-pellet therapy applied in this study is an effective nursing mediation method for pain control as a single therapy and is significant in terms of being able to confirm the intervention timing of the hand pressed-pellet mediation for pain.

In this study, the pre- and postsummation of activities of daily living between the intervention group and the placebo control group were statistically significant. Although the tools used in this study and previous studies on lower back pain that applied hand pressed-pellet were not the same, the level of daily life activities used along with the reduction of pain was improved, which was similar to the results of this study and supported the results. A previous study in which the VAS and the ODI were used with the experimental group and the control group—assessing the effectiveness of acupuncture and placebo acupuncture at the same acupuncture point—also showed a decrease in the VAS and ODI scores [33]. Repeated application of hand pressed-pellet therapy induces long-term neuroplastic changes, reorganizing pain-related neural circuits and reducing central sensitization. This contributes to the restoration of the diminished function of the endogenous pain control system due to chronic pain [16, 17]. The results of applying hand acupressure for six weeks in this study showed improvement in the ability to perform daily living activities in both the experimental and control groups, with the effects being particularly pronounced in the experimental group. This suggests that hand acupressure can lead to functional improvements in addition to reducing pain.

In this study, both the intervention group and the control group showed statistically significant improvement in activities of daily living after six weeks of hand pressed-pellet therapy mediation. The improved results in the control group are also judged to be the result of improved blood circulation according to the principle of acupressure and corresponding therapy with the hand pressed-pellet therapy. In addition, it can be confirmed that the placebo effect appeared because the control group in this study was treated with placebo hand pressed-pellet therapy, and it is evident that the experimental and placebo mediation group structure was effective because no participants knew to which group they belonged. Nevertheless, the hand pressed-pellet therapy applied in this study had the effect of reducing pain as it did in previous studies, enhancing the possibility of utilizing hand pressed-pellet therapy as a nursing intervention for elderly patients with chronic lower back pain. Additionally, hand pressed-pellet therapy is a non-invasive nursing intervention with few side effects, offering the advantage of being safely applicable to elderly patients. From a non-clinical perspective, this study can raise awareness about the major health issue of chronic lower back pain in the elderly and emphasize the importance of non-pharmacological interventions. But, this study has the following limitations. Although efforts were made to minimize these limitations through strategies such as random assignment, the influence of a single mediator and investigator cannot be completely eliminated. Future research needs to separate the roles of mediators and investigators to minimize research bias. Additionally, since there are various types of low back pain, research is needed to subdivide the causes and characteristics of low back pain to verify the effects. Therefore, the results of this study should be interpreted considering these limitations, and it will be necessary to enhance the generalizability of the results through multi-institutional studies in the future.

## Conclusion

This study confirmed that the hand pressed-pellet mediation applied for six weeks was effective in reducing pain intensity, increasing the pain pressure threshold, and improving activities of daily living in elderly people complaining of chronic lower back pain. This study confirmed the effect of nursing mediation by presenting scientific evidence obtained from measuring both subjective and objective indicators of pain to confirm the effectiveness of hand pressed-pellet therapy in the placebo control and the intervention group. This highlights the clinical relevance of hand pressed-pellet therapy as a non-invasive and low-risk nursing intervention for managing chronic lower back pain symptoms and aiding functional recovery in the elderly. The therapy’s safety and minimal side effects make it an advantageous treatment for elderly patients.

## Data Availability

The material and data that support the findings of this study are available from the corresponding author upon request.

## Abbreviations

CLCP: chronic lower back pain
VAS: Visual Analogue Scale
K-ODI: the Korean Owestry Disability Index

## References

[1] World Health Organization (WHO). (2023). Low back pain. Retrieved from April 1, 2024. https://www.who.int/news-room/fact-sheets/detail/low-back-pain/

[2] Ferreira ML et al. Global, regional, and national burden of low back pain, 1990–2020, its attributable risk factors, and projections to 2050: a systematic analysis of the Global Burden of Disease Study 2021. The Lancet Rheumatology. 2023;5(6): e316-e329. 10.1016/S2665-9913(23)00098-X 10.1016/S2665-9913(23)00098-X PMC10234592 37273833

[3] Dagnino AP, Campos MM. Chronic pain in the elderly: mechanisms and perspectives. Front Hum Neurosci. 2022;16. 10.3389/fnhum.2022.736688 10.3389/fnhum.2022.736688 PMC8928105 35308613

[4] Konstantinovic LJ, Foti C, Dragin A, Filipovic S. Factors of disability in geriatric patients with low back pain. Proceedings of the Xi European Congress of the European Federation for Research in Rehabilitation. 2011; 85–86.

[5] Traeger AC et al. Low back pain in people aged 60 years and over. Bmj 376 (2022). 10.1136/bmj-2021-066928 10.1136/bmj-2021-066928 35318211

[6] Hartvigsen J, Hancock MJ, Kongsted A, Louw Q, Ferreira ML, Genevay S (2018). What low back pain is and why we need to pay attention. Lancet.

[7] Foster NE, Anema JR, Cherkin D, Chou R, Cohen SP, Gross DP (2018). Prevention and treatment of low back pain: evidence, challenges, and promising directions. Lancet.

[8] Qaseem A, Wilt TJ, McLean RM, Forciea MA, Robert M, Clinical Guidelines Committee of the American College of Physicians (2017). Noninvasive treatments for acute, subacute, and chronic low back pain: a clinical practice guideline from the American College of Physicians. Ann Intern Med.

[9] Jung G, Kim J (2011). Comparison of conventional medicines and complementary-alternative therapy utilization on musculoskeletal pain. Health Soc Rev.

[10] Yoo T, Yoo (2017). Taewoo’s seogum therapy.

[11] Yang J (2009). The effects of hand acupuncture therapy on pain, ROM, ADL and depression among elders with low back pain and knee joint pain. J Korean Acad Nurs.

[12] Kim Y, Choi S, Kim J (2019). Effects of hand moxibustion and press pellet therapy on low back pain, range of joint movement, and depression. J Korean Acad Comm Health Nurs.

[13] Park H, Yang H (2020). The effect of hand press pellet therapy on arthralgia, ankylosis, and depression in elderly women with knee osteoarthritis. Korean J Rehab Nurs.

[14] Park H, Kim N (2019). Effects of the self-managed seogum therapy among college students with allergic rhinitis. J East-West Nurs Res.

[15] Beresford-Cooke. Carola. Shiatsu theory and practice. Singing Dragon; 2016.

[16] Clement-Jones V, McLoughlin L, Tomlin S, Besser GM, Rees LH, Wen HL (1980). Increased beta-endorphin but not met-enkephalin levels in human cerebrospinal fluid after acupuncture for recurrent pain. Lancet.

[17] T.W. Yoo, Seokum therapy, Koryosujichim, Seoul, 2011.

[18] Ahn NY, Park HJ (2017). Effects of Korean hand acupressure on opioid-related nausea and vomiting, and pain after caesarean delivery using spinal anaesthesia. Complement Ther Clin Pract.

[19] Lee Y, Kim J (2010). The effects of hand acupuncture moxibustion therapy on elders’ shoulder pain, ADL/IADL and sleep disorders. J Korean Acad Comm Health Nurs.

[20] Kim Y, Park H (2020). Effects of hand acupressure on sleep quality and pruritus in patients on hemodialysis. Korean J Adult Nurs.

[21] Jeong D, Park H (2021). Effects of hand press pellet on constipation in patients with breast cancer receiving chemotherapy. Korean J Adult Nurs.

[22] Cohen J (1988). Statistical Power Analysis for the behavioral sciences.

[23] Hyoung HK (2008). Effects of a strengthening program for lower back in older women with chronic low back pain. J Korean Acad Nurs.

[24] Lim N, Lee Y (2003). The effects of Koryo hand-acupuncture on the patients with chronic low back pain. J Korean Acad Nurs.

[25] Gwak S (2021). Healthy hand acupunctures.

[26] Fischer A (1987). Pressure algometry over normal muscles. Standard values, validity and reproducibility of pressure threshold. Pain.

[27] Lee J, Lee J, Shin H, Yoon C, Oh M, Kwon S (2008). Usefulness of electronic pressure algometer in evaluation of pressure Pain threshold in normal Korean adults. J Korean Acad Rehabilitation Med.

[28] Fairbank JC, Couper J, Davies JB, O’Brien JP (1980). The Oswestry low back pain disability questionnaire. Physiotherapy.

[29] Jeon C, Kim D, Kim D, Lee H, Park H (2006). Cross-cultural adaptation of the Korean version of the Oswestry Disability Index (ODI). J Korean Spine Surg.

[30] Kim SK, Park H (2021). The effect of auricular acupressure for chronic low back pain in elders: a randomized controlled study. Holi Nurs Pract.

[31] Kim H (2016). The effects of moxibustion therapy on chronic low back pain, daily living disability and sleep pattern in elderly women. J Korea Acad-Indust Coop Soc.

[32] Lee J, Lee J, Shin H, Yoon C, Oh M, Kwon S (2008). Usefulness of electronic pressure algometer in evaluation of pressure pain threshold in normal Korean adults. J Korean Med Rehab.

[33] Cho YJ, Song YK, Cha YY, Shin BC, Shin IH, Park HJ (2013). Acupuncture for chronic low back pain: a multicenter, randomized, patient-assessor blind, sham-controlled clinical trial. Spine.

